# Evaluation of quality of care in relation to health-related quality of life of patients diagnosed with brain tumor: a novel clinic for proton beam therapy

**DOI:** 10.1007/s00520-018-4557-7

**Published:** 2018-11-27

**Authors:** Ulrica Langegård, Karin Ahlberg, Per Fransson, Birgitta Johansson, Katarina Sjövall, Thomas Bjork-Eriksson, Emma Ohlsson-Nevo, The Proton Care Study Group

**Affiliations:** 1 Institute of Health and Care Sciences, Sahlgrenska Academy, University of Gothenburg, Arvid Wallgrens backe, Box 457, 405 30 Göteborg, Sweden; 2 Department of Nursing, Umeå University, Umeå, Sweden; 3 Cancercentrum, Norrlands University Hospital, Umeå, Sweden; 4 Experimental Oncology, Department of Immunology, Genetics and Pathology, Uppsala University Hospital, Uppsala University, Uppsala, Sweden; 5 Department of Oncology, Skane University Hospital, Scania, Sweden; 6 Department of Oncology, Lund University, Lund, Sweden; 7 Department of Oncology, Institute of Clinical Sciences, Sahlgrenska Academy at University of Gothenburg, Gothenburg, Sweden; 8 The Skandion Clinic, Uppsala, Sweden; 9 Regional Cancer Center West, Gothenburg, Sweden; 10 University Healthcare Research Centre, Faculty of Medicine and Health, Örebro University, Örebro, Sweden

**Keywords:** Quality of care, Health-related quality of life, Proton beam therapy, Radiotherapy, Brain tumor

## Abstract

**Purpose:**

Patients with brain tumors constitute a vulnerable group, and it is important that they receive the highest quality of care (QoC). The study aim was to describe the perceptions of QoC and its association with health-related quality of life in brain tumor patients undergoing proton beam therapy in a newly established clinic.

**Method:**

Data were collected at the start of treatment and after 3 and 6 weeks. Adult patients (≥ 18 years old) with brain tumors (*n* = 186) completed two self-administered questionnaires: a modified Quality from the Patients’ Perspective, which measures perceived reality and subjective importance of care, and the EORTC QLQ-C30. Data were analyzed using parametric and non-parametric statistical tests.

**Results:**

The perceived QoC was highest for treatment information and lowest for dietician and smoking information, whereas interaction with doctors and nurses was rated as the most important aspect of quality of care. Subjective importance ratings were significantly higher than perceived reality ratings for 60% of items. A better global health was moderately correlated with a higher perceived support for fatigue.

**Conclusions:**

A need for quality improvement was identified for several aspects of patient care. Greater symptom distress during the treatment period led to greater perceived importance of symptom support. Ensuring QoC is complex and collaboration with other health care professionals is essential.

**Relevance to clinical practice:**

The clinic could improve QoC regarding information about possible symptoms, adjust care according to patient perceptions of importance, and involve patients in care decisions.

**Electronic supplementary material:**

The online version of this article (10.1007/s00520-018-4557-7) contains supplementary material, which is available to authorized users.

## Introduction

About 238,000 patients are annually diagnosed with malign brain tumor worldwide [1]. In Sweden, approximately 1300 patients are annually diagnosed with a primary brain tumor and approximately 50% of tumors are malignant [2]. Brain tumors affect people of all ages, but commonly occur in individuals aged over 60 years. Initial symptoms are headache, anorexia, nausea, vomiting, seizures, sleeping longer at night, and drowsiness with napping during the day [3]. Most patients also experience fatigue and double vision [4], as well as neurological deficits and cognitive impairment [5]. Personality changes, mood disturbances, or decreased mental capacity and concentration can occur later in the disease trajectory [3, 6]. Most of these symptoms, particularly cognitive impairments, may be amplified during radiotherapy [7–10].

The World Health Organization considers Quality of Care (QoC) a concern because of the large variance in care delivered within and between health care systems, and has identified four health care dimensions: professional management of care, minimal risk of harm to the patient, effectiveness, and patient satisfaction [11]. Donabedian [12], one of the leading developers of the QoC concept, has identified the following QoC indicators: structure, process, and outcome. Wilde et al. [13] consider QoC a multidimensional concept and a measure of patient’s experiences of the quality of the health care encounter; QoC entails both patient perception of the care received and how important different aspects of care are to patients. Health-related quality of life (HRQoL) describes the effects of disease and treatment on physical, psychological, and social well-being, and includes symptom measures [14]. HRQoL is an important endpoint in health care research and is a common patient-reported outcome measure for cancer patients. HRQoL can predict patient perceptions of QoC [15, 16].

In August 2015, the first proton beam therapy (PBT) clinic in Scandinavia, the Skandion Clinic, began treating patients with tumor diseases and one of the first group of patients were patients with malignant or benign brain tumors. The Skandion Clinic has an estimated annual capacity of 1000 patients. The clinic is organized according to a model of distributed competence and shared governance collaboration, in which all clinical experts work closely together with their patients in the regional home clinic [17]. Seven radiotherapy departments connected to university hospitals in Sweden are involved. Patients are prepared for treatment at the home clinic and treatment data including dose distribution plan and immobilization device are transferred to the Skandion Clinic, which is responsible for the delivery of PBT treatment and for clinical evaluations during treatment. Before start of PBT patients are discussed on bi-weekly national treatment video conferences. After completion of PBT, patients are re-referred back to the home clinic for long-term follow-up. During the treatment period (5–6 weeks), most patients stay at a conventional hotel located in the same building as the Skandion Clinic.

Owing to the specific health care needs of patients with brain tumors, it is essential to investigate how patients experience care in relation to HRQoL. This vulnerable group of patients, who often experience cognitive disorders, must travel long distances to receive PBT treatment; therefore, it is important that the clinic provides the highest quality of care. Further, when starting up a new health care organization, it is important to ensure high QoC so that patients feel that their needs have been met. Therefore, the study aim was to describe the patient perspective on QoC and its associations with HRQoL in brain tumor patients undergoing PBT in a newly established PBT clinic.

## Method

### Design

This study is part of ProtonCare, a larger multicenter project assessing the role of proton treatment compared to other modern photon-based radiotherapy techniques. The ultimate purpose of ProtonCare is to investigate patient-reported variables, e.g., short- and long-term symptoms and HRQoL in patients receiving PBT. The present study is a prospective, longitudinal, quantitative study. Ethical approval was obtained from the research ethics committee’s in Gothenburg 2015-07-22 (Dnr 433–15).

### Participants and procedure

A consecutive sample of 216 adult patients (≥ 18 years) diagnosed with a primary brain tumor undergoing PBT at the Skandion Clinic were invited to participate in the study from August 2015 to December 2017. As several colleges were involved in this multicenter study, a few patients may not have been invited to participate in the study. The exclusion criterion was inability to communicate in Swedish. Study information was provided by the first author (UL) by telephone. Written information about the study was sent by mail together with a consent form. All participants provided informed consent. Data were collected in web-based or paper format according to patients’ choice. The electronic questionnaires were available in a database, and an email was sent to participant’s e-mail address at the start of treatment, after 3 weeks and at the end of treatment. Paper questionnaires were distributed to participants by an oncology nurse at the Skandion Clinic. Participants were provided with a prepaid envelope and asked to return the questionnaires by mail. A reminder was send to the e-mail/home address of participants who did not complete/return the questionnaires within 10 days.

## Data collection

Medical data on tumor disease and treatment were collected from patients’ records and demographic data were obtained by a study specific questionnaire.

### Quality from the patient’s perspective

The Quality from the Patient’s Perspective questionnaire (QPP) was used to measure patients’ views of the QoC [18–20]. The instrument evaluates four dimensions of patient perceptions of QoC: medical-technical (MT), physical-technical (PT), identity-oriented (ID), and sociocultural atmosphere (SC). The QPP was developed from interviews using a grounded theory approach; a model of QoC was created to reflect a deeper understanding of the phenomenon. The questionnaire was psychometrically tested [18] and validated by a dimensional analysis using structural equation modeling [20]. The present study used a short version of the QPP used for radiotherapy in outpatient settings, supplemented with a context-specific (CS) dimension that comprised five items. The baseline questionnaire comprised 32 questions (MT = 4 items, ID = 18, SC = 1, CS = 5) and the follow-up questionnaire comprised 43 questions (MT = 5 items, ID = 20 items, SC = 8 items, CS = 5 items). All questions were rated according to the perception of the reality (PR) of the QoC (i.e., “this is what I experienced”; 1 = do not agree to 4 = fully agree) and the subjective importance (SI) of the care (i.e., “this is how important it was to me”; 1 = of little importance to 4 = of greatest importance). Participants could also choose a “not applicable” response alternative. Four additional items on the experience of waiting to start PBT were included in the baseline questionnaire, and one item on the PBT experience was included in the follow-up questionnaire. The scoring options (PR) for these items ranged from 1 (to a very small extent) to 5 (to a very great extent).

### Health-related quality of life

EORTC QLQ-C30 [14] was used to measure HRQOL. It includes five functional scales, three symptom scales, five single item symptoms, and a global quality-of-life (QOL) scale. All scales and single items are transformed to scores ranging from 0 to 100 [21]. For functional scales and global QOL, a higher score suggest better level of functioning, while for the symptoms, a higher score suggest more severe problems.

## Data analysis

Between-group differences were analyzed using Mann–Whitney *U* tests. Within-group changes from baseline to 3 and 6 weeks were analyzed using Wilcoxon signed rank tests. For between-group comparisons, Fisher’s exact test was used for dichotomous variables and the Mantel–Haenszel chi-squared test was used for ordered categorical variables. Since no differences were found between malignant and benign groups, the results are shown for the total population. A mean value was calculated based on each participant’s PR and SI responses to the items. Low values were estimated as below 1.7, medium as 1.7–3.3, and high as 3.4–4.0. Missing values were not imputed and not applicable responses were treated as missing data. Mean values for the dimensions were only calculated if > 50% of the questions had been answered. Cronbach’s alpha coefficients were computed for testing homogeneity. Correlations were rated as low (< 0.30), moderate–high (0.30–0.60), or substantial (> 0.6) [22]. To describe the discrepancy in frequencies, PR scale responses were dichotomized: *do not agree* and *partly agree were* combined into *do not agree*, and *agree to a large extent* and *fully agree* were combined into *fully agree*. SI scale responses were dichotomized: *no or of little importance* and *of some importance* were combined into *low importance*, and *of great importance* and *of the greatest importance* were combined into *great importance.* As QoC correlates with HRQoL [15, 23], we tested whether QPP scores correlated with scores on the QLQ-C30 function scales, global health, the fatigue symptom scale, emotional functioning scale, and the single-symptom insomnia. Statistical analyses were performed using SAS system version 9.3. Reported *p* values are two-tailed, and *p* < 0.05 was considered statistically significant.

## Results

### Demographic, clinical, and health-related characteristics

The response rate was 86% and the final sample comprised 186 participants. There were 53% women and 47% men, and the mean age was 48 years (range 18–85 years). A total of 8% had mandatory education (< 10 years), 46% had high school education, and 40% had university or higher education. The most common occupations were employed (80%), retired with a pension (18%), and student (2%). Most patients had a good performance status: Eastern Cooperative Oncology Group (ECOG) 0–1 and Karnofsky Performance Score (KPS) 80–100%. Characteristics of the study population are shown in Table 1.

**Table 1:** Participant’s demographic information (*n* = 186)

	Number	Percent
Sex		
Woman	98	53
Man	88	47
Age		
Mean (SD)	48 (14)	
18–25	9	4
26–35	31	17
36–45	40	22
46–55	48	26
56–65	30	16
66–75	26	14
76–85	2	1
Civil status		
Single	57	31
Married	129	69
Education		
Mandatory school	15	8
High school	86	46
University or higher education	76	40
Diagnose		
Tumor localization		
C 70: malignant tumor in CNS meningium	4	2
C 71: malignant tumor in the brain	94	51
C 75: malignant tumor in pituitary	2	1
D 18: hemangiom	3	2
D 32: benign tumor in CNS meningium	51	27
D 33: benign tumor in in the brain	12	6
D 35: benign tumor in pituitary	14	8
D 43: uncertain benign tumor in brain or CNS	5	3
D 44: uncertain benign tumor in endocrine glands	1	0
Questionnaires		
Digital format	43	23
Paper format	143	77

### QPP perceived reality

The internal consistency reliability coefficients (Cronbach’s alpha) for the PR subscale ranged between 0.83 and 0.89 at baseline (Table 2). PR results are shown in Table 3 (baseline to 6 weeks) and Supplementary Table 1 (3 to 6 weeks). Medium levels of PR were reported for the four dimensions (mean 2.05–3.21). High PR ratings (> 3.4) were shown for items about treatment information and common symptoms (items 5, 6, and 9) and for doctor and nurse interactions with the patient (items 17–22). Low PR ratings (< 1.6) were shown for items about dietician information and smoking (items 25, 26, and 28). There were significant improvements in PR after 6 weeks only for items about treatment information (item 6), self-care (item 11), symptoms (items 12, 13), doctors’ understanding (item 17), and good information about physical activity (item 24).

**Table 2:** Cronbach’s alphas for Quality From Patient’s Perspective questionnaire scores

Quality from patient perspective	Cronbach alpha for dimension perceived reality	Cronbach alpha for dimension subjective importance	Number of items
Baseline (dimensions)			
Medical-technical	0.85	0.91	4
Physical-technical conditions			0
Identity-oriented	0.89	0.94	18
Socio-cultural atmosphere			1
Context specific	0.83	0.88	5
Follow-up (dimensions)			
Medical-technical	0.68	0.80	5
Physical-technical conditions			0
Identity-oriented	0.91	0.93	20
Socio-cultural atmosphere	0.80	0.89	8
Context specific	0.83	0.80	5

The table shows the Cronbach’s alphas for quality of care ratings on the Quality from the Patient’s Perspective dimensions. Perceived reality and subjective importance ratings are shown at the start of treatment (baseline) and at follow-up (3 and 6 weeks)

**Table 3:** Perceived reality and subjective importance ratings of quality of care from the patient’s perspective

Dimensions	Perceived reality, mean (standard deviation)				Subjective importance, mean (standard deviation)					
	Baseline (*n* = 186)	6 weeks (*n* = 186)	Change from baseline to 6 weeks	*p* value	Baseline (*n* = 186)	6 weeks (*n* = 186)	Change from baseline to 6 weeks	*p* value	*p* = PR and SI difference at baseline	*p* = PR and SI difference: baseline to 6 weeks
Medical-technical competence										
1. I received examinations and treatment within an acceptable waiting time	3.39 (0.80)	3.47 (0.82)	0.103 (0.968)	0.12	3.41 (0.82)	3.30 (0.88)	− 0.086 (0.935)	0.21	0.49	0.02
2. I received effective support for my fatigue when necessary	2.03 (1.06)	2.20 (1.14)	0.056 (1.149)	0.76	2.83 (1.06)	2.89 (1.06)	0.047 (1.119)	0.64	< 0.0001	< 0.0001
3. I received effective support for my sleeping problems when necessary	2.24 (1.18)	2.39 (1.19)	0.032 (1.293)	0.89	2.95 (1.09)	2.84 (1.11)	− 0.179 (1.081)	0.22	< 0.0001	0.001
4. I received effective support for worry and anxiety	2.36 (1.17)	2.30 (1.15)	0.066 (1.181)	0.61	3.10 (0.96)	2.84 (1.12)	− 0.269 (1.050)	0.07	< 0.0001	0.001
Total mean	2.05	2.59	0.097 (0.806)	0.24	3.07	2.96	0.413 (0.921)	0.00		
Identity-oriented approach										
5. I received good information about the preparations (CT scan, fixation, treatment plan)	3.52 (0.66)	3.58 (0.68)	0.051 (0.695)	0.31	3.32 (0.83)	3.45 (0.73)	0.106 (0.836)	0.15	0.01	0.01
6. I received good information about the treatment	3.49 (0.74)	3.64 (0.63)	0.136 (0.634)	0.00	3.40 (0.76)	3.53 (0.67)	0.116 (0.724)	0.04	0.26	0.06
7. I received good information about the different steps in my continued care	3.14 (0.91)	3.15 (0.87)	0.011 (0.953)	0.81	3.38 (0.74)	3.40 (0.75)	0.006 (0.806)	0.93	0.00	< 0.0001
8. I had good opportunity to participate in decisions about my care	2.89 (1.03)	3.01 (0.95)	0.054 (1.052)	0.58	3.11 (0.97)	3.17 (0.90)	0.050 (0.953)	0.56	0.01	0.02
9. I received good information about common symptoms	3.43 (0.75)	3.49 (0.73)	0.045 (0.727)	0.47	3.52 (0.69)	3.52 (0.72)	− 0.023 (0.690)	0.60	0.17	0.58
10. I received good information about the results of examinations and the treatment	3.01 (0.95)	2.94 (1.00)	− 0.056 (0.933)	0.40	3.65 (3.19)	3.42 (0.77)	− 0.006 (0.784)	0.88	< 0.0001	< 0.0001
11. I received good information about self-care (e.g., diet and exercise)	2.46 (1.09)	2.66 (1.06)	0.201 (0.986)	0.01	3.08 (0.97)	3.05 (0.99)	− 0.013 (0.993)	0.86	< 0.0001	< 0.0001
12. I received good information about how to prevent or relieve symptoms	2.64 (1.06)	3.06 (0.91)	0.402 (1.096)	< 0.0001	3.20 (0.84)	3.33 (0.85)	0.113 (0.931)	0.13	< 0.0001	0.001
13. I received good information about how long the symptoms of radiation therapy might last	2.57 (1.10)	3.26 (0.88)	0.669 (1.210)	< 0.0001	3.25 (0.85)	3.44 (0.75)	0.163 (0.793)	0.01	< 0.0001	0.01
14. I received good information about which doctor is responsible for my medical care	2.87 (1.06)	2.88 (1.07)	− 0.028 (1.087)	0.66	3.26 (0.91)	3.37 (0.80)	0.105 (0.943)	0.19	< 0.0001	< 0.0001
15. I received good information about which nurse is responsible for my nursing care	3.25 (0.91)	3.38 (0.94)	0.126 (1.086)	0.10	3.28 (0.89)	3.32 (0.89)	0.053 (0.786)	0.44	0.67	0.54
16. I received good information in response to questions about my oncological treatment	2.80 (1.04)	2.93 (1.02)	0.079 (1.007)	0.33	3.20 (0.92)	3.25 (0.95)	0.027 (0.932)	0.81	< 0.0001	0.001
17. The doctors seemed to understand how I experienced my situation	3.40 (0.74)	3.53 (0.68)	0.132 (0.825)	0.05	3.57 (0.68)	3.54 (0.69)	− 0.006 (0.754)	0.99	0.01	0.001
18. The doctors were respectful towards me	3.70 (0.56)	3.77 (0.50)	0.071 (0.602)	0.13	3.66 (0.58)	3.66 (0.61)	0.012 (0.596)	0.78	0.70	0.01
19. The doctors showed commitment, “cared about me”	3.57 (0.69)	3.65 (0.62)	0.095 (0.758)	0.07	3.63 (0.63)	3.60 (0.66)	− 0.006 (0.622)	0.90	0.29	0.37
20. The nurses seemed to understand how I experienced my situation	3.66 (0.60)	3.73 (0.52)	0.057 (0.623)	0.25	3.59 (0.64)	3.56 (0.67)	− 0.035 (0.685)	0.43	0.22	0.001
21. The nurses were respectful towards me	3.78 (0.47)	3.85 (0.48)	0.051 (0.527)	0.15	3.63 (0.62)	3.63 (0.61)	− 0.012 (0.604)	0.80	0.01	< 0.0001
22. The nurses showed commitment, “cared about me”	3.76 (0.52)	3.83 (0.47)	0.063 (0.514)	0.12	3.63 (0.64)	3.62 (0.63)	− 0.023 (0.639)	0.64	0.04	< 0.0001
Total mean	3.21	3.35	− 0.027 (0.486)	0.50	3.40	3.43	0.065 (0.418)	0.03		
Sociocultural atmosphere										
23. My care was determined by my own requests and needs rather than staff procedures	3.05 (0.94)	3.14 (0.93)	0.059 (0.949)	0.36	3.12 (0.96)	3.25 (0.96)	0.086 (0.928)	0.21	0.49	0.10
Context-specific										
24. I received good information about how physical activity could increase my well-being	2.80 (1.06)	2.95 (1.02)	0.160 (0.996)	0.05	3.18 (0.91)	3.22 (0.95)	0.018 (0.936)	0.68	< 0.0001	0.001
25. I received good information about how I could change my diet if necessary	1.68 (0.92)	1.67 (0.92)	− 0.016 (0.913)	0.83	2.67 (1.06)	2.47 (1.12)	− 0.243 (1.038)	0.02	< 0.0001	< 0.0001
26. I received good information about how I can obtain the support of a dietician if necessary	1.51 (0.92)	1.53 (0.90)	0.054 (0.942)	0.63	2.55 (1.16)	2.28 (1.14)	− 0.396 (1.124)	0.00	< 0.0001	< 0.0001
27. I received good information about how smoking can affect the radiation treatment	2.39 (1.29)	2.39 (1.29)	− 0.127 (0.982)	0.35	2.71 (1.33)	2.44 (1.29)	− 0.216 (1.436)	0.42	0.69	0.94
28. I received good information about how I could get help to stop smoking	1.87 (1.19)	1.82 (1.23)	0.000 (1.181)	1.00	2.65 (1.33)	2.22 (1.28)	− 0.400 (1.501)	0.30	0.00	0.62
Total mean	2.25	2.07	− 0.275 (0.847)	0.09	2.75	2.52	− 0.075 (0.652)	0.31		

The table shows the perceived reality and subjective importance of the quality of care from the patient’s perspective for items on the four dimensions of the Quality from the Patient’s Perspective (QPP) questionnaire. Mean values and standard deviations on the QPP items and dimensions are shown to facilitate comparison with other studies. The *p* values refer to differences tested with the Wilcoxon signed rank test. Statistical significance was assumed at the *p* < 0.05 level

CT computerized tomography, PR perceived reality, SI subjective importance

### QPP subjective importance

The internal consistency reliability coefficients (Cronbach’s alpha) for the SI subscale ranged between 0.88 and 0.94 at baseline. Tables 3 and 4 show the results for SI. The dimension *Identity-oriented approach* was of great importance (mean 3.4). The SI of the other two dimensions was of medium importance (3.07, respectively, 2.75). The items of greatest importance were related to interactions with doctors and nurses and treatment information. No item was rated as of low importance (< 1.7). The importance of item 13 (“I received useful information about how long the symptoms of radiation therapy might last”) showed a significant change, as it was rated as more important after 6 weeks. There was a significant decrease in importance of dietician information after 6 weeks (items 25, 26).

**Table 4:** Responses on the European Organization for Research and Treatment of Cancer Quality of Life Questionnaire (QLQ C-30)

Variable	Baseline (*n* = 186)	3 weeks (*n* = 186)	6 weeks (*n* = 186)	Change from baseline to 3 weeks	*p* value	Change from baseline to 6 weeks	*p* value
Global health status	68.5 (19.0)	65.0 (20.6)	60.7 (20.3)	− 3.92 (14.22)	0.001	− 8.19 (17.55)	< 0.0001
	66.7 (25.0, 100.0)	66.7 (16.7, 100.0)	66.7 (0.0, 100.0)	0.00 (− 83.33, 41.67)		− 8.33 (− 58.33, 66.67)	
	*n* = 186	*n* = 185	*n* = 177	*n* = 185		*n* = 177	
Physical functioning	85.9 (17.5)	84.4 (18.8)	84.4 (17.5)	− 1.95 (10.45)	0.03	− 2.06 (12.39)	0.05
	93.3 (13.3, 100.0)	93.3 (13.3, 100.0)	93.3 (20.0, 100.0)	0.00 (− 66.67, 26.67)		0.00 (− 73.33, 26.67)	
	*n* = 186	*n* = 185	*n* = 177	*n* = 185		*n* = 177	
Role functioning	67.1 (33.3)	65.7 (33.2)	60.2 (33.1)	− 1.98 (28.00)	0.38	− 8.19 (31.81)	< 0.0001
	66.7 (0.0, 100.0)	66.7 (0.0, 100.0)	66.7 (0.0, 100.0)	0.00 (− 100.00, 100.00)		0.00 (− 100.00, 100.00)	
	*n* = 186	*n* = 185	*n* = 175	*n* = 185		*n* = 175	
Emotional functioning	74.5 (22.0)	82.4 (20.2)	82.3 (21.1)	7.16 (18.43)	< 0.0001	6.26 (19.69)	< 0.0001
	75.0 (0.0, 100.0)	83.3 (0.0, 100.0)	91.7 (0.0, 100.0)	8.33 (− 50.00, 83.33)		8.33 (− 50.00, 66.67)	
	*n* = 186	*n* = 185	*n* = 177	*n* = 185		*n* = 177	
Cognitive functioning	79.9 (21.6)	81.4 (21.2)	77.5 (23.4)	0.901 (17.867)	0.63	− 3.77 (18.59)	0.001
	83.3 (0.0, 100.0)	83.3 (16.7, 100.0)	83.3 (16.7, 100.0)	0.000 (− 50.000, 50.000)		0.00 (− 50.00, 50.00)	
	*n* = 186	*n* = 185	*n* = 177	*n* = 185		*n* = 177	
Social functioning	74.4 (26.9)	73.4 (27.9)	73.0 (26.8)	− 1.71 (23.22)	0.35	− 2.54 (26.79)	0.18
	83.3 (0.0, 100.0)	83.3 (0.0, 100.0)	83.3 (0.0, 100.0)	0.00 (− 100.00, 50.00)		0.00 (− 100.00, 100.00)	
	*n* = 186	*n* = 185	*n* = 177	*n* = 185		*n* = 177	
Fatigue	32.1 (24.4)	34.1 (24.7)	39.9 (26.3)	2.94 (16.55)	0.02	9.04 (20.77)	< 0.0001
	33.3 (0.0, 100.0)	33.3 (0.0, 100.0)	33.3 (0.0, 100.0)	0.00 (− 44.44, 55.56)		0.00 (− 44.44, 66.67)	
	*n* = 186	*n* = 185	*n* = 177	*n* = 185		*n* = 177	
Nausea	6.11 (17.11)	7.75 (16.12)	7.16 (15.35)	2.16 (14.06)	0.05	2.45 (16.00)	0.02
	0.00 (0.00, 100.00)	0.00 (0.00, 100.00)	0.00 (0.00, 100.00)	0.00 (− 50.00, 83.33)		0.00 (− 83.33, 100.00)	
	*n* = 186	*n* = 185	*n* = 177	*n* = 185		*n* = 177	
Pain	14.9 (23.1)	17.7 (23.7)	19.0 (25.5)	3.87 (19.07)	0.01	5.84 (22.34)	0.01
	0.0 (0.0, 100.0)	16.7 (0.0, 100.0)	0.0 (0.0, 100.0)	0.00 (− 50.00, 83.33)		0.00 (− 66.67, 83.33)	
	*n* = 186	*n* = 185	*n* = 177	*n* = 185		*n* = 177	
Dyspnea	17.0 (24.7)	18.7 (27.3)	20.9 (26.3)	1.81 (22.78)	0.26	4.92 (21.40)	0.001
	0.0 (0.0, 100.0)	0.0 (0.0, 100.0)	0.0 (0.0, 100.0)	0.00 (− 66.67, 66.67)		0.00 (− 33.33, 100.00)	
	*n* = 186	*n* = 185	*n* = 177	*n* = 184		*n* = 176	
Insomnia	24.1 (30.0)	27.7 (30.1)	28.2 (32.1)	3.24 (26.04)	0.08	4.14 (28.35)	0.08
	0.0 (0.0, 100.0)	33.3 (0.0, 100.0)	33.3 (0.0, 100.0)	0.00 (− 100.00, 100.00)		0.00 (− 66.67, 100.00)	
	*n* = 186	*n* = 185	*n* = 177	*n* = 185		*n* = 177	
Appetite loss	9.95 (20.80)	12.6 (21.6)	15.1 (24.9)	3.24 (20.00)	0.03	6.40 (24.03)	0.001
	0.00 (0.00, 100.00)	0.0 (0.0, 100.0)	0.0 (0.0, 100.0)	0.00 (− 66.67, 100.00)		0.00 (− 66.67, 100.00)	
	*n* = 186	*n* = 185	*n* = 177	*n* = 185		*n* = 177	
Constipation	6.98 (19.29)	8.83 (19.04)	12.2 (22.9)	2.16 (19.23)	0.09	5.84 (23.51)	0.001
	0.00 (0.00, 100.00)	0.00 (0.00, 100.00)	0.0 (0.0, 100.0)	0.00 (− 100.00, 66.67)		0.00 (− 66.67, 100.00)	
	*n* = 186	*n* = 185	*n* = 177	*n* = 185		*n* = 177	
Diarrhea	7.33 (17.23)	7.39 (16.28)	9.04 (18.29)	0.721 (16.651)	0.56	2.82 (19.09)	0.05
	0.00 (0.00, 100.00)	0.00 (0.00, 66.67)	0.00 (0.00, 66.67)	0.000 (− 66.667, 66.667)		0.00 (− 66.67, 66.67)	
	*n* = 186	*n* = 185	*n* = 177	*n* = 185		*n* = 177	
Financial difficulties	20.4 (28.3)	15.9 (26.3)	18.5 (30.5)	− 4.17 (20.04)	0.001	− 1.51 (26.07)	0.17
	0.0 (0.0, 100.0)	0.0 (0.0, 100.0)	0.0 (0.0, 200.0)	0.00 (− 100.00, 66.67)		0.00 (− 66.67, 200.00)	
	*n* = 186	*n* = 184	*n* = 177	*n* = 184		*n* = 177	

The table shows the mean values (standard deviations) at baseline and total changes over time at 3 weeks and 6 weeks (for both malignant and benign brain tumor subgroups). For continuous variables, means (standard deviations)/medians (min, max)/*n* are shown. For comparisons over time, the Wilcoxon signed rank test was used for continuous variables. High functional scores represent better HRQoL and high symptom scores indicate more severe symptoms. Statistical significance was assumed at the *p* < 0.05 level

### Quality of care

There was a discrepancy between patients’ experiences with their care (PR) and how important they perceived the care (SI); SI scores for 55% of the items were significantly higher than PR scores. These differences were found on the dimensions medical-technical competence, (items 2–4), identity-oriented (items 7–8, 10–14, 16–17), and context-specific (items 24–26). Of these 15 significant items, 60% concerned information or consequences related to symptoms such as fatigue, sleeping problems, worry and anxiety, and participation in care decisions (Table 3). Supplementary Table 2 shows the discrepancy in ratings of items about symptoms. On 44% of the items, patients perceived that the SI increased from baseline to 6 weeks. An increase in symptom experience during the treatment period led to an increase in the SI of symptom support.

### Health-related quality of life

Global health status, physical functioning, role functioning, and cognitive functioning significantly decreased over time, whereas emotional functioning significantly improved from baseline to 6 weeks. The symptoms of fatigue, nausea, and pain increased after 6 weeks. There were also significant increases on the single items: dyspnea, insomnia, appetite loss, constipation, and diarrhea (Table 4). There were no significant differences between the subgroups on the function scales, except for cognitive function. Thus, there were significant differences between the groups on some of the symptom scales. In the benign group, symptoms increased significantly: nausea (4.71–7.48, *p* ≤ 0.001), pain (16.9–25.0, *p* ≤ 0.001), dyspnea (14.1–20.1, *p* ≤ 0.001), insomnia (23.1–29.9, *p* = 0.05), appetite loss (9.80–18.4, *p* ≤ 0.001), and diarrhea (3.92–8.12, *p* = 0.04). Several characteristics of the benign group may explain the significant changes over time: the age of this group was significantly higher (*p* < 0.001), it contained significantly more women (*n* = 55 [65%], *p* ≤ 0.001), and the education level was significantly lower (*p* ≤ 0.001), not shown in the table.

### Quality of care in relation to health-related quality of life

**Fig. 1:**
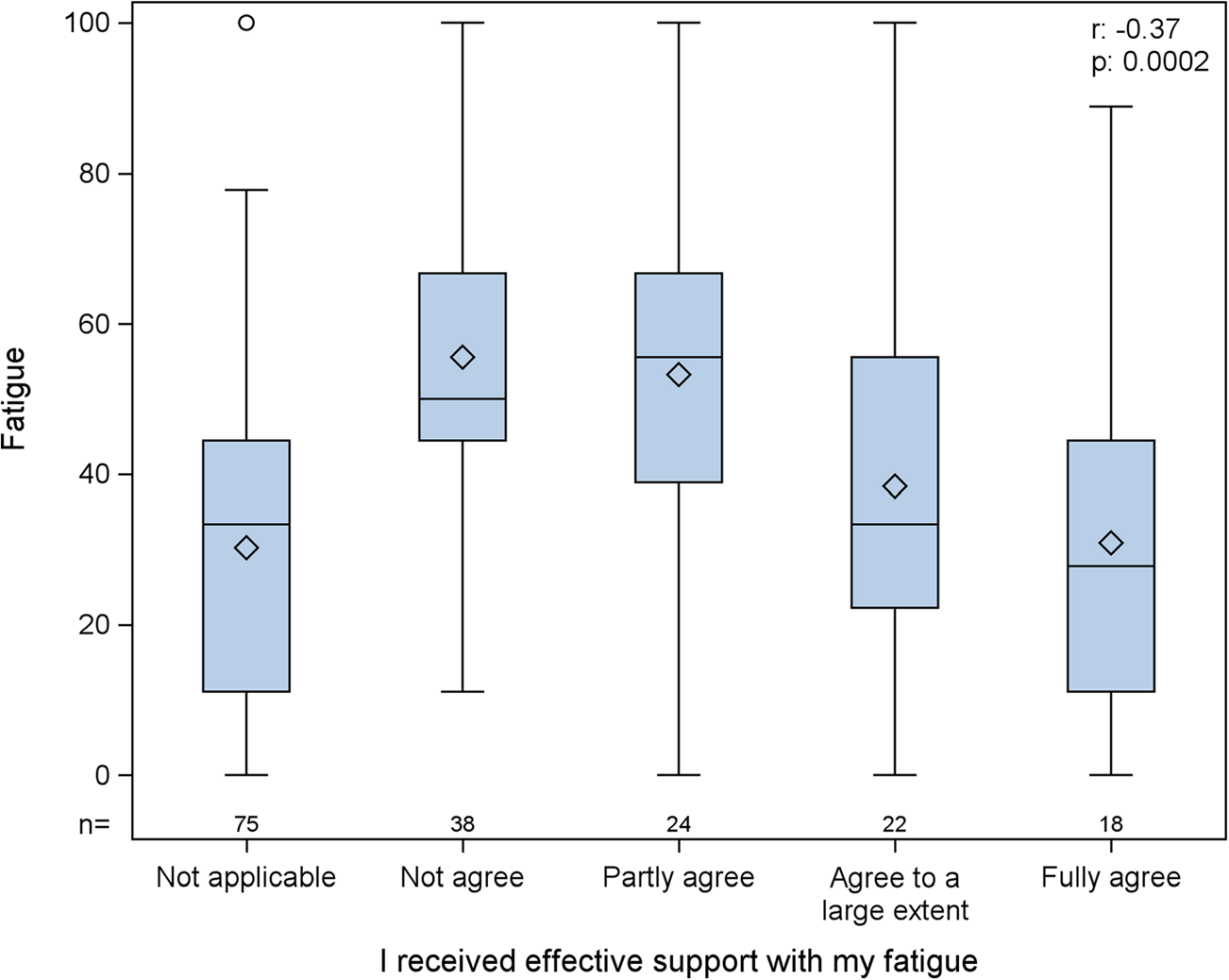
Score distributions and correlation for experienced fatigue and patients’ perception of effective support for the symptom. *x* axis QPP and *y* axis EORTC QLQ-C30. There were 75 patients who responded “not applicable,” in the end of treatment, despite experiencing fatigue. QPP Quality from the Patient’s Perspective questionnaire, EORTC QLQ-C30 European Organization for Research and Treatment of Cancer Quality of Life Questionnaire

Patients who experienced a high level of global health reported a high level of effective support with fatigue, understanding from the doctor, and receipt of information about common symptoms (*r* = 0.30–032, *p* = 0.001 to < 0.001). There were no significant correlations between QPP SI ratings and HRQoL. QLQ-C30 fatigue scores correlated negatively with the QPP item *support with fatigue* at baseline (*r* = − 0.32, *p* ≤ 0.001) and at 6 weeks (*r* = − 0.37, *p* ≤ 0.0002). Thus, patients who had high levels of fatigue perceived that they did not receive effective support from the health care staff. Figure 1 shows the distributions and correlation between experienced fatigue and patients’ perceptions of receiving effective support for the symptom in the end of treatment. The figure shows that 75 patients responded not applicable, although they had experienced fatigue.

## Discussion

To our knowledge, this is the first study to investigate QoC in relation to HRQoL in a PBT clinic. The main finding was that more than 50% of the care was perceived as of inadequate quality, especially regarding support with symptom management. Reported QoC improved during the treatment period but not sufficiently.

### General reported QoC

The QoC was generally reported as moderate for all dimensions, although treatment-related information and doctor/nurse interaction with patients were rated highly. The findings indicated that multiple care domains required improvement. Patients perceived a lower QoC in relation to fatigue, insomnia, and worry/anxiety. Patients felt that they did not receive adequate support in symptom management; these results are consistent with previous research [24, 25]. Symptoms of brain tumor patients can be alleviated by support and attention from health care professionals [26, 27]. Ratings for the context-specific dimension indicated a need for improvement regarding nutrition and physical activity. Large improvements in these could be obtained by providing contact with a dietician a physiotherapist and other activities favoring exercise during the treatment period. Previous QoC studies have shown that information provided to patients is often insufficient [24, 28, 29]. It is thus important to further develop information provision to meet the special needs of patients with brain tumors. One way to reduce the risk that patients miss information is to adopt clear routines on national basis; e.g., brochures and electronic aids for information provision and for constant following up the information patients have received. It can be assumed that this is especially important for a clinic like the Skandion Clinic based on shared governance and distributed competence and where many of the patients receive their treatment far away from their homes and families.

### QoC dimensions

The ID dimension expresses patients’ desire for care from qualified caregivers with the knowledge and empathic skills to encounter the patient as a unique person [13]. On this dimension, we found that patients experienced high QoC for items about respect, commitment, and empathy; however, 50% of items (measuring information about the different steps in continued care, opportunity to participate in the care, and good information about self-care and how to prevent symptoms) showed inadequate QoC. Ratings for this dimension reflected perceptions of high levels of humanity and empathic skills, but indicated that professional knowledge aspects required quality improvement. Facilitation of patient participation in care and the support of patients’ symptom management are important nursing skills that require improvement [24]. To help patients participate more in their self-care, the clinic could develop collaborations with professionals such as dieticians and physiotherapists and arrange common group activities to enhance knowledge about nutrition and physical activities. Changing clinic routines requires effort, but is necessary to meet patient needs.

Our current findings add important knowledge to previous work and confirm results by Janda et al. [30]. This study shows that patients expect and need support through the treatment period and the symptoms that brain tumor patients experience require advanced care from the health care organization, including objective assessment of neuropsychological functioning and education and psychosocial support. The ID dimension also included information about which physician is responsible for medical care. Patients reported low QoC concerning physician continuity, participation in decisions about care, which doctor was responsible for medical care, and how to obtain information about the oncological treatment. Ratings of low QoC in these areas may be because although the Skandion Clinic provided the PBT, the home clinic had the main responsibility for post-treatment care. These results indicate that the model of distributed competence used [17] does not fully meet the patients’ care needs. Treatment of a brain tumor requires advanced health care, and availability, proactive and flexible support, professionalism, and empathic skills are essential [26]. Radwin [31] has argued that oncology patients’ definition of excellent care includes professional knowledge, continuity, attentiveness, coordination, partnership, individualization, and a caring nursing approach, in which nurses express concern and are nurturing. This study identified areas of satisfactory QoC as well as areas needing improvement. Patients reported high QoC for the dimension sociocultural atmosphere, indicating that patients were satisfied and no improvements are needed. We suggest that increased information about symptoms and symptom management would improve care.

### Relationship between QoC and HRQoL

The relationship between HRQoL and QoC remains unclear. Previous studies have reported contradictory results, according to whether functional or symptom scales were used, highlighting the complexity of the patient’s interpretation of QoC. Previous research shows that poor perceived global health is strongly associated with dissatisfaction with aspects of care such as lack of technical and interpersonal skills and insufficient information provided by caregivers [16]. There is also evidence that QoC is associated with better clinical outcomes, such as improved physical function and quality of life [16]. An interesting finding in the present study was that almost 40% of patients gave not applicable responses on the QPP item support with fatigue, despite reporting fatigue on the QLQ-C30. This was an unexpected finding and may indicate that the question was misunderstood, or that patients felt that it did not apply to QoC, even though they experienced fatigue.

The present findings must be interpreted in the light of the limitations of the study. The use of multiple tests may have affected the results; therefore, they should be interpreted with caution. However, the results are comparable with findings from previous studies. One possible limitation is the use of both electronic and paper data collection methods, although research suggests that there is little or no difference in reliability between such methods [32, 33]. It is possible that patients’ gratitude and loyalty to the health care staff prevented them making negative evaluations and promoted positive evaluations, as shown in Staniszewska’s studies on patient evaluation of the QoC [34, 35]. It is also possible that patients felt grateful to be able to receive PBT, and thus were likely to give positive responses.

One clear strength of the study is that it described patients’ perspectives of the whole treatment period and changes in QoC were followed over time. A second strength is the high response rate and that a researcher has had the main responsibility and was always available for the participants.

## Conclusion

A need for quality improvement was identified for 60% of the items about information or consequences related to symptoms such as fatigue, sleeping problems, worry and anxiety, and participation in care decisions. Increased symptom distress during the treatment period leads to more importance of focusing on symptom support; therefore, the patients must receive appropriate information if they are to participate in their care. QoC is a complex issue and must be developed in close collaboration with other health care professionals.

## Implications

The present findings have important implications for health care professionals. The clinic could improve its procedures regarding information about symptoms that may occur and should adjust care according to brain tumor patients’ perception of its importance, as well as involve patients in decisions about care.

## Electronic supplementary material

ESM 1 (DOCX 21 kb)

ESM 2 (DOC 39 kb)
