# Supplementary material for: Evaluation of quality of care in relation to health-related quality of life of patients diagnosed with brain tumor: a novel clinic for proton beam therapy
Source: Support Care Cancer. 2018 Nov 27;27(7):2679–91. doi: 10.1007/s00520-018-4557-7 (PMC6541566; doi:10.1007/s00520-018-4557-7)
Supplement: Supplementary file 1 — (DOCX 21 kb) [file 520_2018_4557_MOESM1_ESM.docx]

| Supplementary Table 1. Perceived reality and subjective importance ratings of quality of care from the patient’s perspective: changes from 3 to 6 weeks | | | | | | | | |
| --- | --- | --- | --- | --- | --- | --- | --- | --- |
| Perceived reality  Mean (standard deviation) | | | | Subjective importance  Mean (standard deviation) | | | | |
| DIMENSIONS | 3 weeks (n = 186) | 6 weeks (n = 186) | p-value | 3 weeks (n = 186) | 6 weeks (n = 186) | p-value | p-value PR and SI difference  3 weeks | p-value PR and SI difference  6 weeks |
| MEDICAL-TECHNICAL COMPETENCE |  |  |  |  |  |  |  |  |
| 1. During my time at the unit, I perceived that the staff had the necessary skills as far as I could tell | 3.69 (0.63) | 3.79 (0.52) | 0.03 | 3.69 (0.55) | 3.67 (0.62) | 0.66 | 0.87 | 0.001 |
| IDENTITY-ORIENTED APPROACH |  |  |  |  |  |  |  |  |
| 2. I usually met the same doctor during the treatment period | 2.25 (1.19) | 2.20 (1.14) | 0.58 | 2.88 (1.09) | 2.87 (1.10) | 0.97 | <0.0001 | <0.0001 |
| 3. I usually met the same nurse during the treatment period | 3.34 (0.85) | 3.29 (0.91) | 0.57 | 3.20 (0.90) | 3.14 (0.92) | 0.42 | 0.06 | 0.03 |
| SOCIOCULTURAL ATMOSPHERE |  |  |  |  |  |  |  |  |
| 4. Collaboration between the units worked well | 3.35 (0.92) | 3.40 (0.82) | 0.61 | 3.50 (0.63) | 3.47 (0.71) | 0.37 | 0.05 | 0.27 |
| 5. I was given the opportunity to talk with the healthcare staff in private | 3.64 (0.77) | 3.72 (0.67) | 0.26 | 3.42 (0.81) | 3.44 (0.83) | 1.00 | 0.001 | <0.0001 |
| 6. I felt that the reception staff treated me with respect | 3.87 (0.42) | 3.93 (0.28) | 0.04 | 3.44 (0.77) | 3.45 (0.72) | 0.45 | <0.0001 | <0.0001 |
| 7. My relatives were treated well | 3.73 (0.66) | 3.83 (0.51) | 0.03 | 3.56 (0.66) | 3.56 (0.66) | 0.64 | 0.001 | <0.0001 |
| 8. It was easy to reach the unit via telephone | 3.22 (0.96) | 3.32 (0.87) | 0.16 | 3.45 (0.75) | 3.40 (0.75) | 0.87 | 0.02 | 0.31 |
| 9. It was easy to get an appointment with the doctor | 3.50 (0.75) | 3.60 (0.65) | 0.07 | 3.50 (0.68) | 3.47 (0.67) | 0.99 | 0.90 | 0.05 |
| 10. It was easy to get an appointment with the nurse | 3.56 (0.73) | 3.61 (0.69) | 0.48 | 3.48 (0.75) | 3.41 (0.77) | 0.30 | 0.51 | 0.001 |
| Total mean | 3.55 | 6.63 |  | 3.5 | 3.5 |  |  |  |
| The table shows the perceived reality and subjective importance of the quality of care from the patient’s perspective for items measured after 3 and 6 weeks on the four dimensions of the Quality from the Patient’s Perspective (QPP) questionnaire. Means and standard deviations of the QPP dimensions and summary scores are shown to facilitate comparison with other studies and are based on rank. The p-values refer to differences tested with the Wilcoxon signed rank test. Statistical significance was assumed at the p<0.05 level. PR: perceived reality; SI: subjective importance. | | | | | | | | |
