# Supplementary material for: Evaluation of quality of care in relation to health-related quality of life of patients diagnosed with brain tumor: a novel clinic for proton beam therapy
Source: Support Care Cancer. 2018 Nov 27;27(7):2679–91. doi: 10.1007/s00520-018-4557-7 (PMC6541566; doi:10.1007/s00520-018-4557-7)
Supplement: Supplementary file 2 — (DOC 39 kb) [file 520_2018_4557_MOESM2_ESM.doc]

# Evaluation of quality of care in relation to health-related quality of life in brain tumor patients receiving proton beam therapy

Supportive Care in Cancer

| Supplementary Table 2. Ratings of quality of care from the patient’s perspective and discrepancy between patient’s perception of the information provided and the importance of the information | | | | | | |
| --- | --- | --- | --- | --- | --- | --- |
|  | Perceived reality  *Do not agree* | | | Subjective importance  *Great importance* | | |
| DIMENSIONS | Baseline (n = 186) | 3 weeks (n = 186) | 6 weeks (n = 186) | Baseline (n = 186) | 3 weeks  (n = 186) | 6 weeks  (n = 186) |
| MEDICAL-TECHNICAL COMPETENCE |  |  |  |  |  |  |
| 1. I received effective support for my fatigue when necessary | 66 (68.0%) | 64 (62.2%) | 63 (60.6%) | 62 (66.7%) | 69 (74.2%) | 64 (66.7%) |
| 2. I received effective support for my sleeping problems when necessary | 48 (55.8%) | 53 (61.6%) | 42 (51.2%) | 60 (73.1%) | 51 (66.3%) | 47 (61.9%) |
| 3. I received effective support for worry and anxiety | 47 (55.9%) | 50 (61.7%) | 43 (56.6%) | 63 (78.8%) | 45 (65.2%) | 43 (64.2%) |
| 4. I received good information about self-care (e.g., diet and exercise) | 82 (47.4%) | 78 (45.9%) | 79 (46.5%) | 123 (73.3%) | 126 (74.6%) | 122 (73.0%) |
| 5. I received good information about how to prevent or relieve symptoms | 78 (43.1%) | 70 (39.3%) | 50 (28.3%) | 136 (77.3%) | 145 (82.4%) | 152 (86.8%) |
| 6. I received good information about how long the symptoms of radiation therapy might last | 85 (46.9%) | 63 (35.0%) | 35 (19.7%) | 145 (81.0%) | 149 (83.3%) | 159 (90.4%) |
| 7. I received good information about how physical activity could increase my well-being | 70 (39.3%) | 67 (37.9%) | 55 (31.6%) | 135 (77.6%) | 138 (78.8%) | 138 (80.2%) |
| 8. I received good information about how I could change my diet if necessary | 120 (79.5%) | 116 (81.7%) | 113 (81.9%) | 84 (59.2%) | 61 (46.9%) | 66 (51.6%) |
| 9. I received good information about how I can obtain the support of a dietician if necessary | 113 (81.9%) | 111 (85.3%) | 104 (83.8%) | 72 (55.4%) | 47 (41.6%) | 44 (40.4%) |
| The table shows the discrepancy between the perceived reality (PR) and the subjective importance (SI) of information provided for selected items. The PR scale was dichotomized to combine *do not agree* and *partly agree* into *do not agree.* The SI scale was dichotomized to combine *of great importance* and *of* *the greatest importance* into *great importance.* | | | | | | |

Ulrica Langegård, Institute of Health and Care Sciences, Sahlgrenska Academy, Gothenburg University,

[ulrica.langegard@gu.se](mailto:ulrica.langegard@gu.se)
